# Desaturases: Structural and mechanistic insights into the biosynthesis of unsaturated fatty acids

**DOI:** 10.1002/iub.2671

**Published:** 2022-09-18

**Authors:** Michela Cerone, Terry K. Smith

**Affiliations:** ^1^ Biomedical Sciences Research Complex University of St Andrews St Andrews Scotland

**Keywords:** biotechnological advances, desaturase, fatty acid, polyunsaturated fatty acids

## Abstract

This review highlights the key role of fatty acid desaturases in the synthesis of naturally occurring, more common and not unsaturated fatty acids. The three major classes of fatty acid desaturases, such as acyl‐lipid, acyl‐acyl carrier protein and acyl‐coenzyme A, are described in detail, with particular attention to the cellular localisation, the structure, the substrate and product specificity and the expression and regulation of desaturase genes. The review also gives an insight into the biocatalytic reaction of fatty acid desaturation by covering the general and more class‐specific mechanistic studies around the synthesis of unsaturated fatty acids Finally, we conclude the review by looking at the numerous novel applications for desaturases in order to meet the very high demand for polyunsaturated fatty acids, taking into account the opportunity for the development of new, more efficient, easily reproducible, sustainable bioengineering advances in the field.

## 1 INTRODUCTION

Fatty acids (FAs) are major structural components of all biological membranes and therefore they have essential roles in modulating the structure and activity of membrane‐associated protein/enzymes. FAs are a source of a large variety of lipid signalling molecules and they are involved in numerous biological pathways such as cell adaptation and survival, modulation of ion channels, endocytosis and exocytosis, pathogen defence and cell signalling.^1^, ^2^ FAs are components of a large and diverse variety of structural molecules including oil, waxes, sterols, glycerophospholipids, sphingolipids, triacylglycerols and numerous biologically functional molecules, such as those involved in lipid metabolism control, signalling, inflammation, immune response and cell division.^3^ Moreover, polyunsaturated fatty acids (PUFAs) have been shown to display a fundamental role in human health, particularly essential polyunsaturated fatty acids (EPUFA).^4^ This class of PUFAs, including ω‐3 and ω‐6 FAs, has been identified as a group of important molecules that intervene in the prevention of inflammation, diabetes, immune system disorders, cardiovascular system, brain development and mental health.^5^ Furthermore, very long‐chain polyunsaturated fatty acids (VLC‐PUFA) have been shown to have essential functions both at the structural and functional cellular level, because they are the major constituents of ceramides and sphingolipids in membrane microdomains.^1^ The large diversity of unsaturated fatty acids (UFAs) is determined by the vast repertoire of enzymes over all three kingdoms of life, namely desaturases. These desaturases play a key role in the de novo biosynthesis of FAs in all biological systems, working in a concerted and regulated manner with elongase enzymes.^6^ A wide number of genes encoding for desaturases have been identified as being responsible for the synthesis of a large variety of UFAs, in both eukaryotes and prokaryotes.^7^ These enzymes, involved in the biosynthesis of UFAs and UFA‐like products, have developed in the course of species' evolution from archaebacteria to mammals. This has led to the identification of different subfamilies of desaturases, but with similar structures despite functional diversity.^8^, ^9^ This aspect is fundamental to predict the complex relationship between the genes encoding for desaturases, their expression regulation, their protein structure, their substrate selectivity, their biocatalytic mechanism and the final chemical structure of the UFA products that are synthesised.^7^ With this in mind, our review will try to give a general insight into structures, activities, substrates and products of different classes of desaturases, whilst highlighting the fundamental role that desaturases play in the regulation of the biosynthesis of UFAs.

## 2 THE ROLE OF FATTY ACID DESATURASE ENZYMES AND THEIR CLASSIFICATION

**FIGURE 1:**
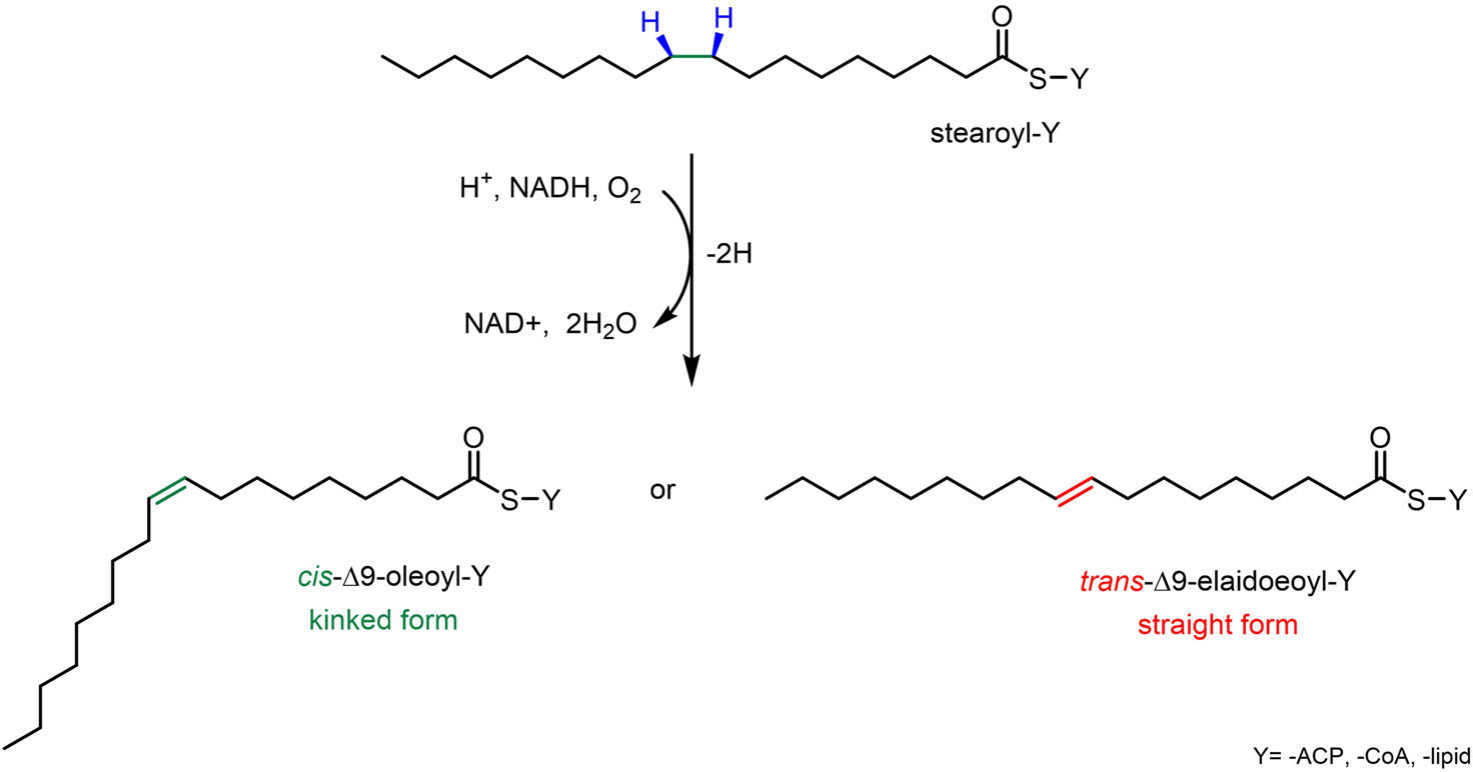
General biocatalytic reaction scheme of the stereospecific and regioselective (blue highlights) insertion of a double bond on the acyl chain of FAs either in *cis* (green highlights) or *trans* (red highlights) conformation catalysed by desaturase enzymes. The scheme also shows how the insertion of the double bond in *cis* causes a kink in the acyl chain, contrarily to *trans* double bonds that maintain the linear acyl chain. Y is ‐ACP or ‐CoA or ‐lipid

FA desaturases are enzymes able to perform dehydrogenation reactions by converting a single bond between two carbon atoms (C—C) to a double bond (C=C) on the acyl chain of a FA molecule in a stereospecific manner.^10^ (Figure 1) The reaction of desaturation is therefore a highly energy‐demanding process of hydrogen abstraction from a methylene group on the acyl carbon chain to obtain UFAs.^11^ (Figure 1) This biocatalytic reaction normally requires an aerobic condition to occur in the majority of the organisms, with the exception of some prokaryotes which are able to synthesise UFAs anaerobically.^11^, ^12^ The desaturation of fatty acids takes place by recruiting molecular oxygen, which is activated into the catalytic site of the enzyme, and by using two electrons to finally give UFAs and two molecules of water as the final products.^11^, ^12^ (Figure 1).

**FIGURE 2:**
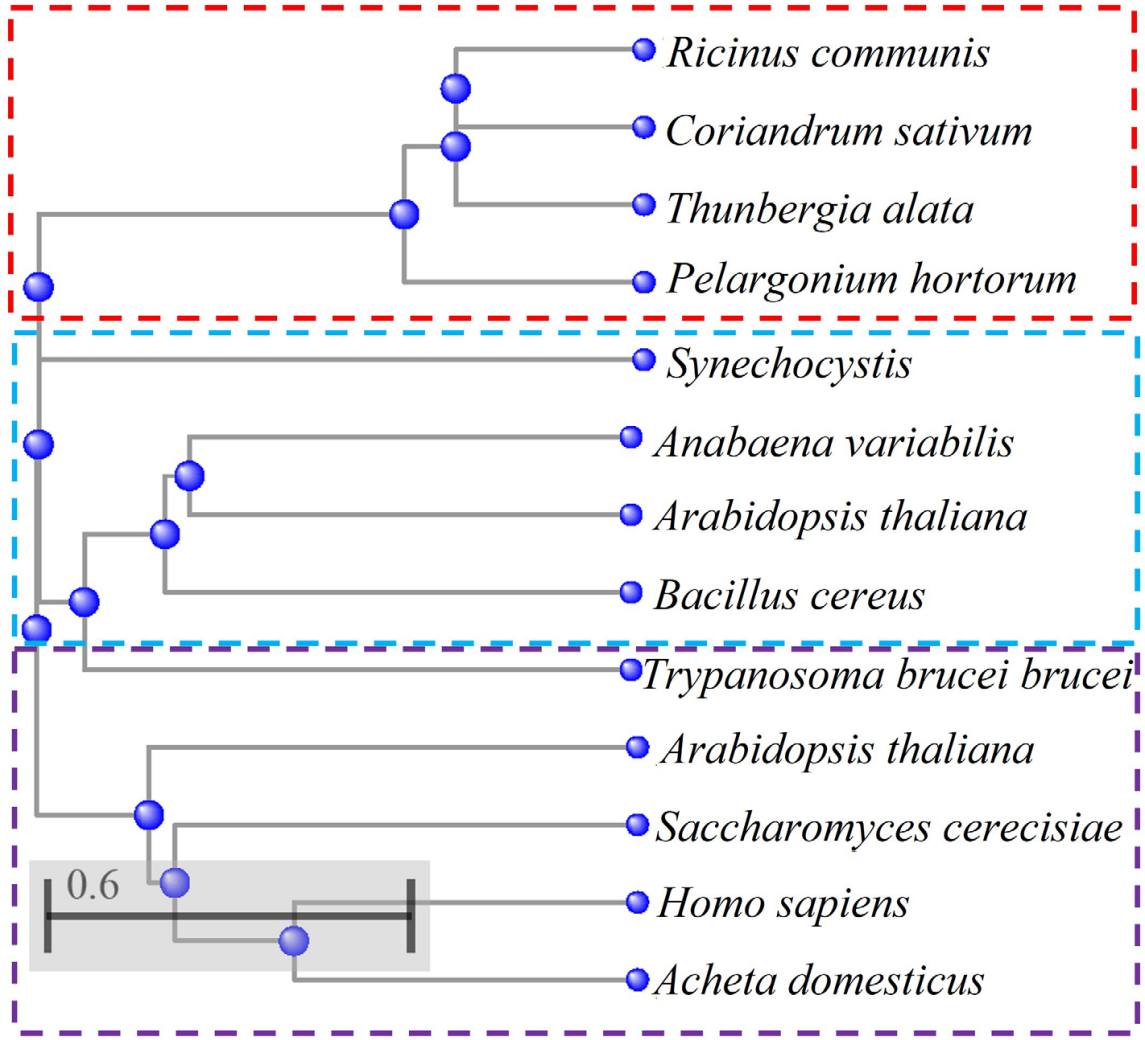
The phylogenetic tree shows the result of a COBALT‐NCBI alignment of FA desaturase protein sequences from various organisms. The tree shows the three classes of desaturase: acyl‐ACPdesaturases from *R. communis* (P22337), *C. sativum* (P32063.1), *T. alata* (Q41510), *P. hortorum* (Q40879) (dashed red box); Acyl‐lipid desaturases from *Synechochystis* (Q53551), *A. variabilis* (Q79F72), *A. thaliana* (P46310), *B. cereus* (A0A164RBC8) (dashed light blue box); Acyl‐CoA desaturases from *T. brucei* (Q38AQ3), *A. thaliana* (A0A1P8AN77), *S. cerevisiae* (P21147), *H. sapiens* (O00767), *A. domesticus* (cricket) (B7SB91) (dashed purple box). The protein sequences were obtained from the UniProtKB database.

The biochemical relevance of the reactions of FAs desaturation is linked to the fundamental role in the regulation of lipid membrane homeostasis. Desaturases are able to insert the appropriate number of double bonds in the acyl chains of FA molecules, according to the level of fluidity required in the membrane for processes such as cell adaptation, activation of various biochemical mechanisms and membrane‐bound protein functions. The control over the lipid bilayer fluidity is particularly determined by the naturally higher level of *cis*‐UFAs, whose configuration confers higher fluidity by forming kinks within the FA chain, compared to the *trans*‐UFAs, which instead give a compact and rigid structure.^13^ (Figure 1) It is via this mechanism, that many organisms are able to balance the transition of the lipid bilayer from gel‐solid to liquid‐crystalline phase in response to chemical and physical alterations, in order to maintain an ideal environment for the cellular processes to take place.^14^, ^15^ Thus, desaturases have been shown to play a primary role in the modulation of the structural equilibrium of the cell membranes by specific and regulated biosynthesis of UFAs.^16^ Although all desaturases utilise a very similar reaction mechanism, it is possible to identify three major classes of fatty acids desaturase from prokaryotes to eukaryotes: acyl‐ACP, lipid‐ACP and acyl‐CoA desaturases.^17^ (Figure 2).

**FIGURE 3:**
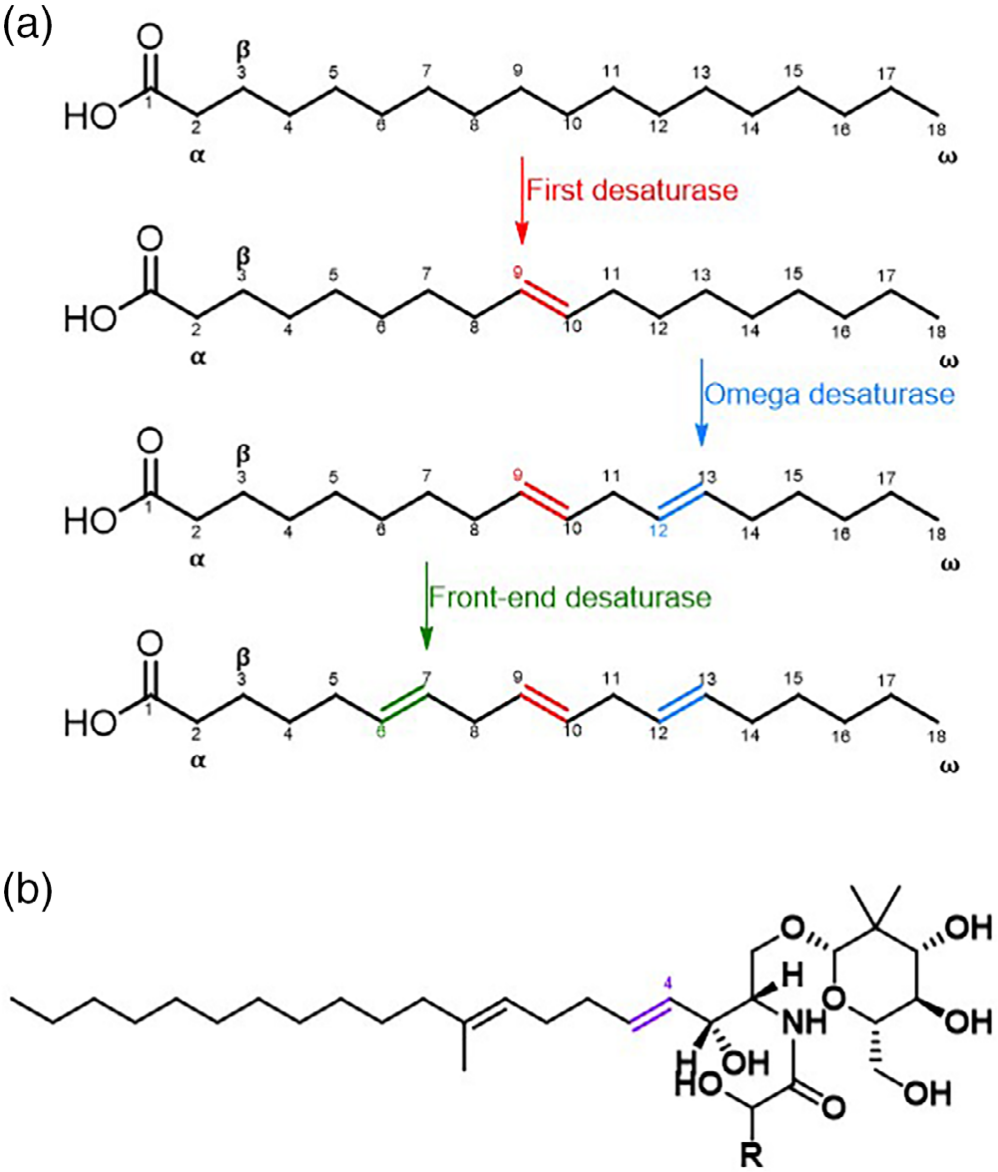
Panel (a) and (b) are schematic representations of the fatty acids and the various positions of the insertion of the double bond by the various type of desaturases. In particular, panel (a) shows examples of UFA products of first desaturase (red highlights), omega (or methyl‐end) desaturase (blue highlights), front‐end desaturase (green highlights); and panel (b) depicts the product of Sphingolipid‐∆4 desaturases (purple highlights).

These three classes can be distinguished in terms of electron carrier system used, solubility and substrate specificity. Thus, in terms of the oxidation reaction, acyl‐lipid and acyl‐ACP desaturases have been shown to use ferredoxin as electron donors, compared to acyl‐CoA desaturases, which utilise the participation of a cytochrome *b5*‐like system, sometimes also used by some acyl‐lipid desaturases.^18^, ^19^ The most represented desaturases belong to the acyl‐ACP class of soluble desaturases and they are localised in the stroma of plastids of higher plants.^20^ They are able to insert a double bond into FAs esterified to an ACP.^20^ The acyl‐lipid desaturases are in contrast membrane‐bound enzymes associated with the endoplasmic reticulum, the chloroplast membrane of plants and the plasmatic and thylakoid membranes of cyanobacteria.^21^ They are able to insert a double bond into FAs that are in a glycerolipid‐esterified form.^21^ Yeasts, fungi and animal cell systems mainly account for the presence of acyl‐CoA desaturases integral membrane proteins associated with the endoplasmic reticulum, whose fatty acid substrates are esterified to CoA.^22^ The differences in the products that they are able to synthesise suggest that each family of desaturases have individually diverged within subfamilies (Figure 2). Thus, each family of desaturases can be further classified into three subfamilies according to the position of the C on the acyl chain of a fatty acid molecule, where the double bond is inserted. Particularly, it is possible to identify 4 groups: (i) First desaturase, which introduces the very first double bond onto the carbon alkyl chain (i.e., ∆9 desaturases) (Figure 3a); (ii) Omega desaturase, which inserts a double bond between a pre‐existing one and the methyl end of the fatty acid chain (i.e., ∆12 and ∆15) (Figure 3a); (iii) Front‐end desaturase, which introduces a double bond between an existing double bond and the carboxylic group (i.e., ∆4, ∆5, ∆6 desaturases) (Figure 3a); (iv) Sphingolipid‐∆4 desaturases, which inserts double bond on fatty acid acyl chain of sphingolipids (Figure 3b).^23^, ^24^, ^25^

## 3 ACYL‐ACP DESATURASES: LOCALISATION, ENZYME STRUCTURE AND SUBSTRATE SPECIFICITY

**FIGURE 4:**
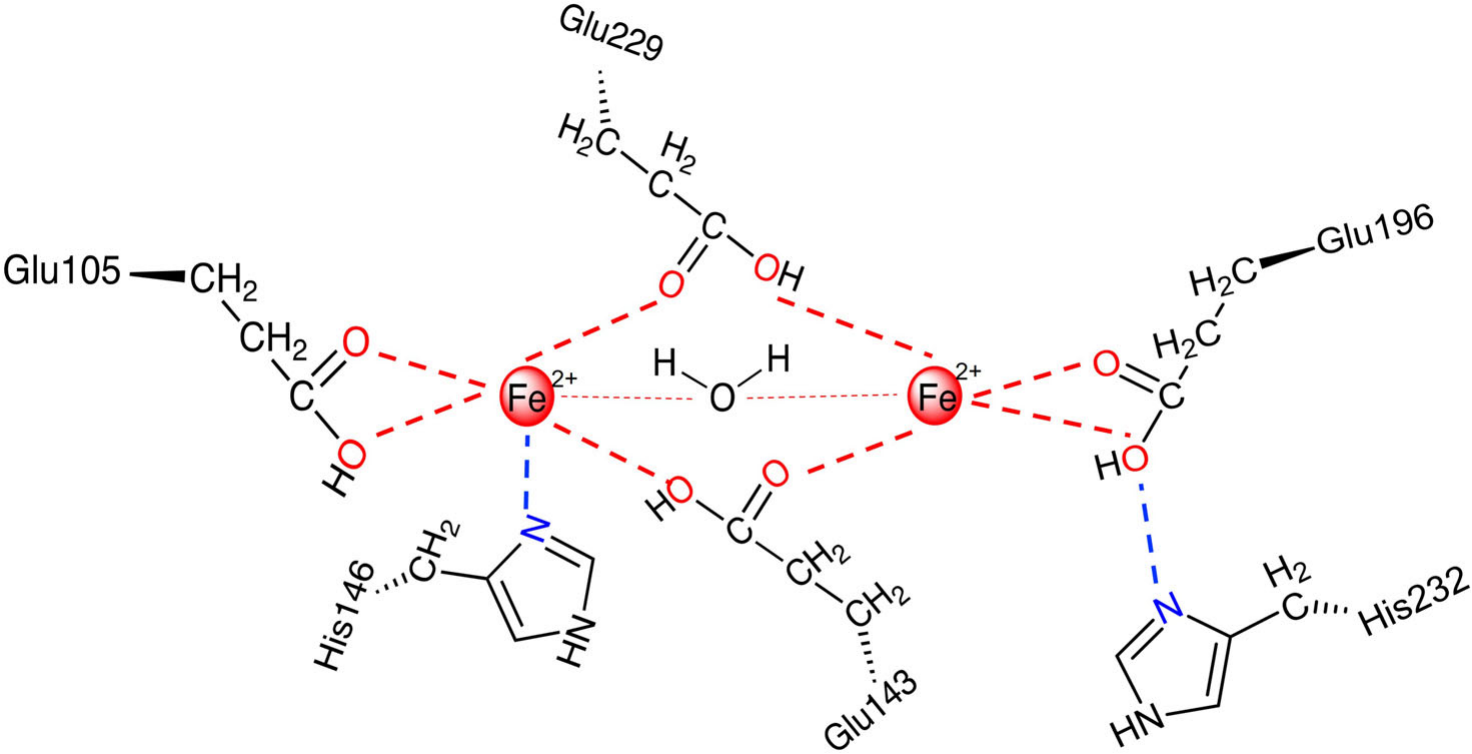
The figure is a simplified representation of the di‐iron catalytic active site in the stearoyl‐ACP desaturase homodimer. The red circles highlight the two irons in their reduced form (Fe^2+^‐Fe^2+^) present in the active site, which are coordinated with glutamate and histidine residues and a molecule of water. This is fundamental for the activity of the desaturases—J. Biol. Chem. 284, 18559–18563(2011).^32^

Acyl‐ACP enzymes are a class of soluble desaturases, which have been found exclusively in higher plants' plastids, where the de novo biosynthesis of PUFAs takes place (Figure 2).^17^ The first acyl‐ACP desaturase was described to be a stearoyl‐ACP desaturase and it was identified by Nagai and Bloch in *Euglena gracilis*.^26^ This enzyme was found to be able to convert a stearate molecule (C18:0), connected to an ACP protein via a thioester link to a pantetheine group, into oleate (C18:1).^27^ The double bond is inserted in a stereospecific manner, in the *cis* configuration, between the C‐9 and the C‐10 of the FA acyl chain forming a ∆^9^‐oleate 18:1 UFA.^27^ After the first identification of stearoyl‐ACP desaturase, others were then reported to be present in the seeds of *Ricinus communis* ^20^ and safflowers embryos, showing the same activity.^28^ (Figure 2) Together with this class of common stearoyl‐ACP desaturase, some others have been identified as unusual acyl‐ACP desaturase. Particularly, they have been found to differ in the chain length of the substrate and the position of the double bond inserted on the acyl chain.^29^ Among those, Δ4‐16:0‐ACP, Δ9‐14:0‐ACP and Δ6‐16:0‐ACP desaturases were isolated respectively from *Coriandrum sativum*, *Pelargonium hortorum*, *Thunbergia alata*.^30^ (Figure 2) The high solubility of this class of desaturases has allowed the isolation, crystallisation and solution of the protein structure. This allows the opportunity for structure‐based studies to understand the substrate binding and specificity and the catalytic reaction mechanism. The crystal structure of Δ9‐stearoyl‐ACP desaturase from *R. communis* has revealed a wide range of important information about the enzyme structure and insights into the binding modes and biocatalytic reaction, which have not been fully exploited yet.^31^ This enzyme is a homodimer of around 40 kDa constituted of four alpha helices with a catalytic binuclear iron active site inserted deep in the middle of this protein. (Figure 4).

More specifically, there are two pairs of antiparallel helices, which create a link with the catalytic iron ions, in their reduced form (Fe^2+^‐Fe^2+^), in a symmetrical manner: one of the iron centres coordinates with a glutamate residue (Glu105) in a bidentate fashion, and with an N atom, belonging to a histidine residue (His146). (Figure 4) In the same way, the second iron centre is coordinated with another glutamate (Glu196) and histidine (His232) in a bidentate and monodentate manner respectively. (Figure 4) another two glutamate residues (Glu143 and Glu229) create a bridge between the two iron centres and interact as ligands for each of the two iron ions.^32^ (Figure 4) Another interaction in the di‐iron site is determined by the presence of a water molecule that weakly coordinates with both iron ions.^32^ The portion of the active site that interacts with the FA substrate shows the presence of a deep and hydrophobic pocket. This cavity extends from the surface of the protein passing through the di‐iron centre, where its bent shape accommodates the gauche conformation of the lipophilic FA acyl chain, therefore, determining the insertion of the double bond in the preferred *cis* configuration.^32^ The regioselectivity has been further investigated by comparing the crystal structures obtained for 18:0‐Δ9‐stearoyl‐ACP desaturase from *R. communis* and 16:0‐Δ9‐stearoyl‐ACP desaturase from ivy, after performing amino acids mutations.^33^ The results of the crystallographic studies suggested that the selection of the carbon chain length is due to the properties of amino acids at the bottom of the cavity. The presence of a different level of a hindrance with the amino acid side chains can enlarge or restrict the cavity maintaining the same electron properties. This leads to an equally efficient desaturation process.^33^ Furthermore, the active site of acyl‐ACP desaturase contains an essential threonine residue that, being a poor electron donor, facilitates the expected hydrogen abstraction from the FA substrate, whereas its replacement has shown to diminish the known catalytic activity.^34^ It is important to underline at this point, that the synthesis of UFAs in higher plants is driven by the presence of this class of ubiquitous desaturase, particularly by Δ9‐stearoyl‐ACP desaturases: once oleate has been synthesised from stearate by stearoyl‐ACP desaturases, the 18:1 substrate can start the cascade to produce PUFAs such as ω6‐18:2 and ω3‐18:3.^35^ Thus, thanks to the presence of these specific classes of EPUFAs, plants are able to finely regulate the level of fluidity of their membrane and activate defence responses in order to adapt to any environmental changes.^36^

## 4 ACYL‐LIPID DESATURASES: LOCALISATION, ENZYME STRUCTURE AND SUBSTRATE SPECIFICITY

**FIGURE 5:**
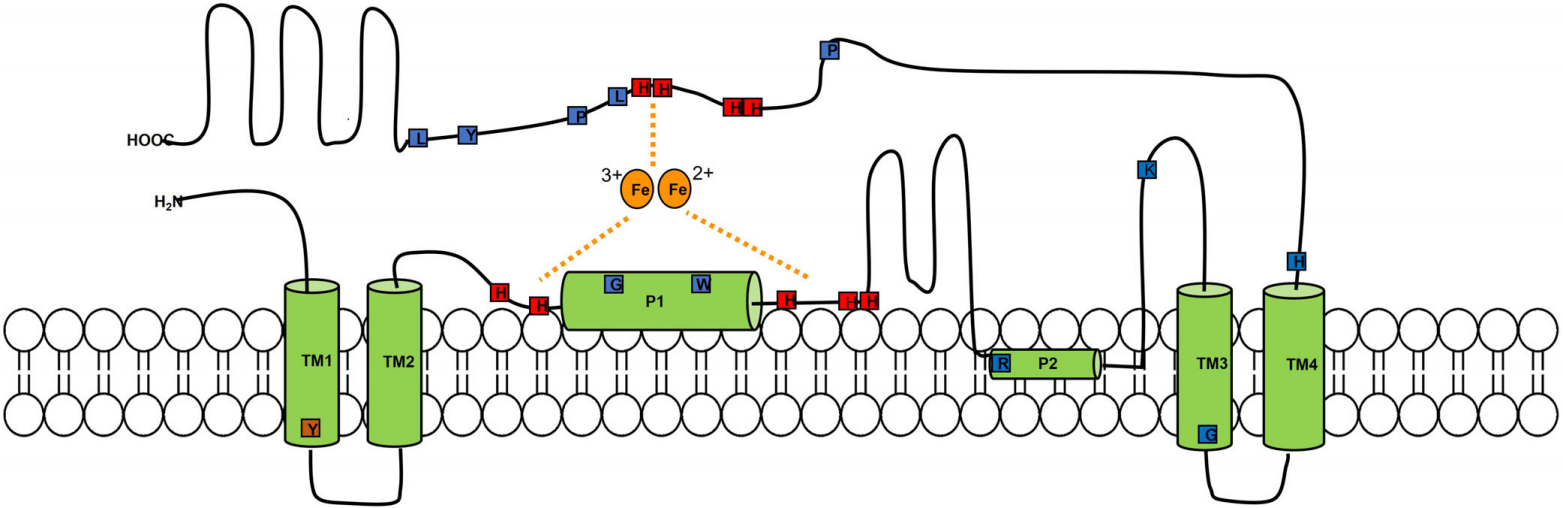
Schematic representation of the topological model of the acyl‐lipid membrane‐bound desaturases. The green cylinders represent the transmembrane domains (TM). The small and coloured boxes highlight the conserved histidine motifs (red) and other essential residues (blue, brown) important for the orientation of the protein in the membrane, the coordination with the di‐iron centre and the substrate selectivity.^42^

Acyl‐lipid desaturases are a class of membrane‐bound desaturases, which are found in the membranes of the endoplasmic reticulum in higher plants and cyanobacteria (Figure 2), where they interact exclusively with the FAs that constitute the side chains of glycerolipids, phospholipids and sphingolipids.^37^ In cyanobacteria acyl‐lipid desaturases play an important role in the process of sequential desaturation in the synthesis of PUFAs: the first step consists of the insertion of a double bond on the C‐9 of stearic acid (18:0), linked to the C‐1 of the glycerolipid, to form oleic acid (Δ^9^‐18:1).^38^ This is the first UFA of the cascade: a series of acyl‐lipid desaturases then intervene sequentially to form Δ^9,12^‐18:2, Δ^6,9,12^‐18:3 and Δ^6,9,12,15^‐18:4.^38^ These reactions are catalysed by three different acyl‐lipid desaturases. They are very specific for the substrate, which is always a fatty acyl chain esterified to the C‐1 of glycerolipid molecule, and strictly selective for the position where the insertion of the double bond on the acyl chain takes place.^38^ Among these enzymes Δ9‐, Δ12‐, ω3‐ and Δ6‐ acyl‐lipid desaturases have been isolated from different strains of cyanobacteria and specifically from *Synechocystis, Synechococcus, Anabaena variabilis*.^39^ (Figure 2) Others such as Δ12‐ and ω3 acyl‐lipid desaturases have been found in the chloroplast of *Arabidopsis thaliana*.^40^ (Figure 2) Extensive studies have been carried out around the relationship between protein structure and reaction mechanism for acyl‐lipid desaturases because of their important role in remodelling the membrane fluidity in response to temperature variation mostly in cyanobacteria, but also in plants.^41^ The similarities found in the sequence and topology of these proteins have allowed the identification of a structural model with conserved amino acid residues and key regions for the activity of acyl‐lipid desaturases.^42^ In particular, the accepted structural model, obtained from the crystal structure of a *B. cereus* acyl‐lipid desaturase, shows the presence of four transmembrane domains (TM 1‐4), two peripheral helices (P1‐2), cytosolic N‐ and C‐ termini and highly conserved histidine boxes.^42^ (Figure 5).

A key tyrosine residue (Tyr40) in the first transmembrane domain was found to be fundamental for the desaturases to anchor and accurately orientate in the lipid bilayer.^42^, ^43^ (Figure 5) In fact, tyrosine is a bulky and amphiphilic amino acid that, when present in a transmembrane domain, tends to stretch perpendicularly into the membrane pointing away from the hydrophobic core.^42^, ^44^ (Figure 5) According to this established phenomenon, this conserved tyrosine seems to arrange the first transmembrane domain in a way that permits the entrance of the acyl chain of the substrate in the active site's cavity, by pulling the core towards the cytosol side.^42^ (Figure 5) The cytosolic domain of the protein, placed between the next two adjacent transmembrane domains, is important to bind to the substrate and to bring the acyl chain in closer proximity to the active site, assuring the stereospecificity and regioselectivity of the desaturation.^42^ (Figure 5) The protein also contains three highly conserved histidine clusters which play an essential role in coordination to the di‐iron centre in the active site for the catalytic activity of acyl‐lipid desaturases, as all the others belonging to this family of enzymes.^42^, ^45^ (Figure 5).

## 5 ACYL‐COA DESATURASES: LOCALISATION, ENZYME STRUCTURE AND SUBSTRATE SPECIFICITY

**FIGURE 6:**
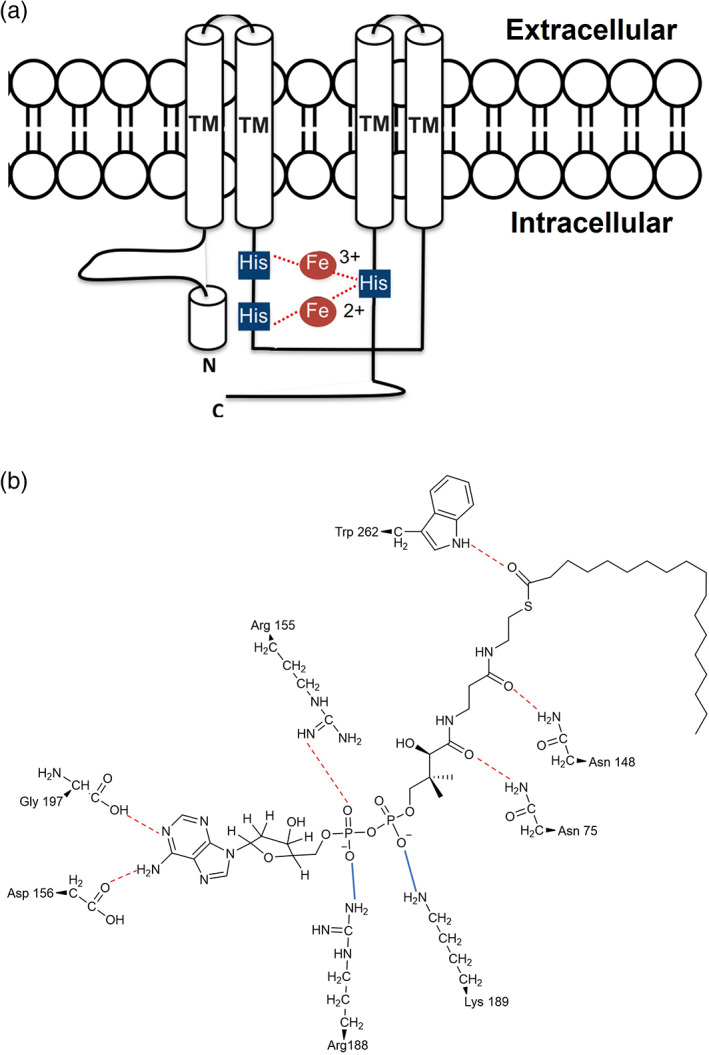
Panel (a) is a schematic representation of the topological model of the acyl‐CoA membrane‐bound desaturases. The white cylinders represent the transmembrane domains (TM). The small blue squares highlight the conserved histidine boxes, that coordinate to the di‐iron centre and determine the correct orientation of the protein in the membrane and interactions with the substrate in a selective and stereospecific manner. Panel (b) shows the interactions between the amino acid residues of the active site and the stearoyl‐CoA substrate. The red dotted lines highlight the H‐bonds. The blue lines highlight the electrostatic interactions.^55^

Acyl‐CoA desaturases are integral membrane proteins found in both eukaryotes and prokaryotes (Figure 2). They are highly represented in endoplasmic reticulum membranes of animals, fungi and yeast and in plastid membranes of plants (Figure 2). In prokaryotes, instead, they are localised in the plasma membrane.^46^ This class of fatty acids desaturases is the most diffuse among different species and includes examples of first, methyl‐end and front‐end desaturases. Thus, they play a very fundamental role not only in the synthesis of UFAs but also in PUFAs and EPUFAs, which are essential biomolecules for membrane biology and signalling processes in all living organisms.^35^ Acyl‐CoA desaturases participate in the sequential cascade of desaturations, in concert with elongation processes, to allow the de novo formation of a large variety of PUFAs. In plants, as we have seen already in the previous sections, once 18:0‐ACP has been efficiently converted into ∆^9^‐18:1‐ACP, and incorporated into the glycerolipids of the chloroplast and endoplasmic reticulum membranes, acyl‐lipid desaturases are able to insert a second and a third double bond, synthesising the ω‐6‐18:2 and ω‐3‐18:3.^47^ Mammals are also able to convert 18:0 to a ∆^9^‐18:1, by using ∆9‐acyl‐CoA desaturases, but they lack ω‐3 and ∆12‐desaturases for the biosynthesis of EPUFAs.^48^ In fact, the presence of a ∆6‐desaturase in the endoplasmic reticulum, and a series of elongases in the same location, allow the production of VLC‐PUFAs, although only by acquiring the starting materials from external sources of PUFAs.^49^ Exceptionally, Nature provides microorganisms such as nematodes, yeasts, microalgae and kinetoplastids, which possess highly specialised machinery to produce a very large variety of PUFAs de novo, meaning there is a wide range of acyl‐CoA desaturases.^50^, ^51^, ^52^, ^53^ (Figure 2) The biochemical importance of these enzymes, and their evident potential for the development of biotechnological advances in producing PUFAs, have meant extensive studies on the structure–function relationship. This has been made particularly challenging by the insolubility of these enzymes.^35^ The few crystal structures obtained and some proposed topology models of Acyl‐CoA‐desaturases, such as ∆12/9‐acyl‐CoA desaturases from cricket,^54^ have revealed the presence of strongly conserved four transmembrane domains and three histidine boxes, respectively important for the orientation of the active site and for the coordination to the di‐iron catalytic centre.^16^, ^17^ (Figure 6a) A very recent study on the structure of a mammalian Δ9‐acyl‐CoA desaturase has confirmed the already established structure of this class of enzyme and has given more details around the protein's structure, in light of the interaction with its substrate stearoyl‐CoA.^55^ This integral membrane protein, found in the lipid bilayer of the endoplasmic reticulum, accounts for four transmembrane alpha helices with a cytoplasmic domain above, which forms a cap at the top. (Figure 6a) The transmembrane domains (TM) are connected by two loops and a polypeptide that stretches towards the cytoplasm of the organelle.^55^ The cytoplasmic region is constituted by 10 alpha helices, which play an important role in the process of coordination to the di‐iron centre, through three highly conserved histidine residues (His120, His125, His269).^55^ In particular, one of the two iron ions is coordinated through the nitrogens to five of the histidine residues, and the second one with four of them.^55^

Substrate recognition seems to be determined by hydrophobic and electrostatic interaction with polar amino acid residues.^55^ Moreover, the CoA head group of the substrate seems to play an important role during the phase of recognition of the substrate and of the regioselectivity of the reaction, by interacting with amino acid residues on the cytoplasmic domain of the desaturase.^55^ (Figure 6b) In particular, the adenosine group of the substrate interacts via H‐bond with the side chain of an arginine (Arg155) and an aspartate (Asp156), and with the carbonyl group of a glycine (Gly197) residue from the second helix of the cytoplasmic domain.^55^ (Figure 6b) Then, the diphosphate of the substrate electrostatically interacts with the side chain of another arginine (Arg188) and a lysine (Lys189). (Figure 6b) Finally, the pantothenate group of the substrate connects with side chains of two asparagine (Asn75 and Asn148) via H–bond^55^ (Figure 6b), The carbonyl group of the acyl chain of the fatty acid substrate forms an H‐bond with the indole group of a tryptophan (Trp262) (Figure 6b). Once these interactions have been established, the acyl chain is then guided into the hydrophobic tunnel, where a pronounced kink determines the selective double bond addition in the pro‐R‐*cis* configuration.^55^ In addition to that, the orientation and the proximity of the di‐iron centre of the two carbons on the acyl chain, where the double bond is inserted, enhances the regioselectivity of the desaturation.^55^ This study further elucidated a structure‐mechanism model on which all acyl‐CoA‐desaturases enzymes seem to rely for their efficient biocatalytic reaction towards the biosynthesis of PUFAs.

## 6 MECHANISM OF DESATURASES REACTIONS

**FIGURE 7:**
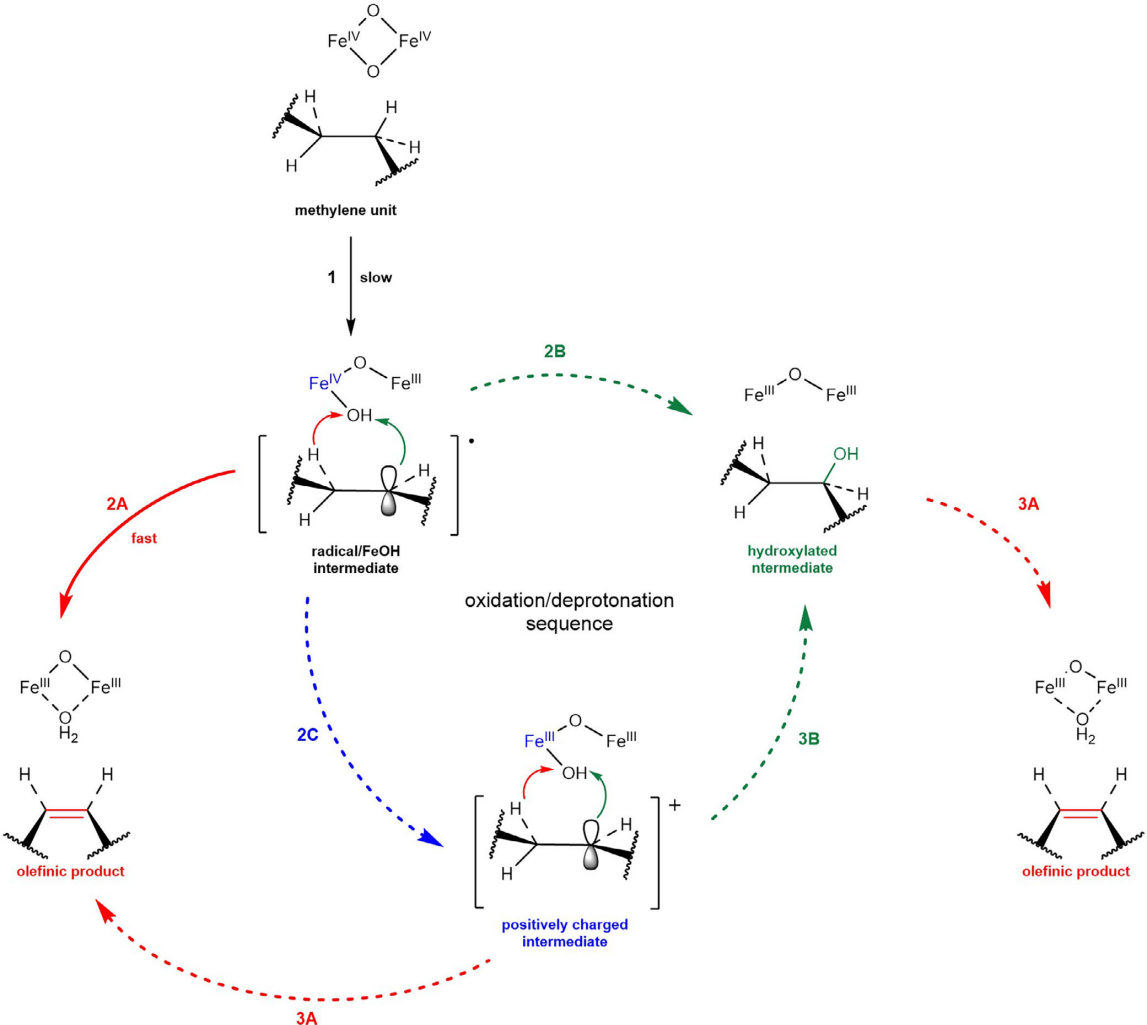
General catalytic mechanism of desaturation of fatty acids (2A and 3A, red) and the interconnection with potential hydroxylation reaction (2B and 3B, green; 2C, blue). The dashed arrows (2C, 2B, 3B, 3A) refer to proposed/hypothetical mechanisms and reaction steps^11^

In sight of the structure of the different desaturases described in the previous sections, what can be said about the mechanism by which desaturase enzymes play their role in the biosynthesis of UFAs? As stated earlier in this review, desaturation is an oxygen‐dependent dehydrogenation of FA acyl chains, which is an ubiquitous biotransformation that produces a large variety of oxidised lipids, which play key roles in biological processes.^11^ The general reaction mechanism for FA desaturases has been extensively studied: a mechanistic model for desaturase‐mediated dehydrogenation has been defined by structural similarities and/or identity between different desaturase families.^11^ The high valent di‐iron centre, present in active sites of all desaturases, has been defined to be the catalytic core of the reaction of dehydrogenation of FAs. (Figure 7) The established model describes the biochemical reaction as characterised by two kinetically different steps. (Figures 7, 1 and 2a) The initial one is considered to be the slow C‐H activation on the methylene unit on the acyl chain. This activation produces a short‐lived C‐centred radical/FeOH reactive intermediate. (Figures 7, 1 slow) Then, this reactive specie goes through a fast deprotonation which consists of a pro‐R‐selective‐*syn* dehydrogenation, giving an olefinic product and an iron‐bound molecule of water. (Figure 7, 2a fast).

**FIGURE 8:**
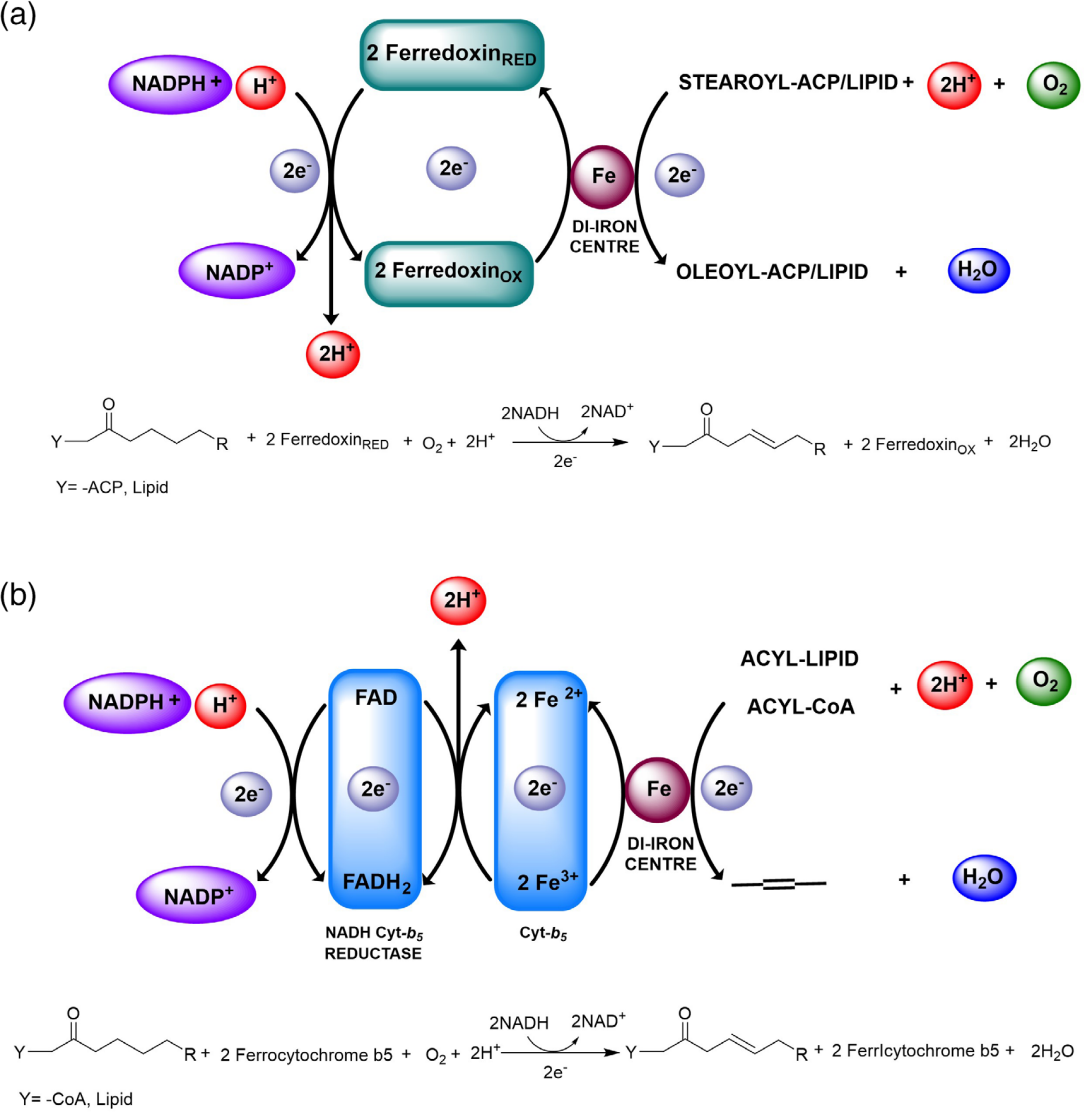
Schematic representation of the electron transport mechanism that takes place in the desaturation of FAs, and stochiometry of the desaturation reaction, According to the type of desaturases there are two possible electron donor systems involved in the catalytic process: ferredoxin (a) and cytochrome‐*b*_*5*_ like unit (b). The di‐iron centre can either be in Fe^*2*+^‐Fe^*2*+^ or Fe^*3*+^‐Fe^*2*+^ as reported in Guy, J. E. et al., J. Biol. Chem., 284,^28^ 18559–18563 (2011)^32^

The last step could alternatively consist of the formation of an alcohol intermediate, through a rebound reaction with FeOH formed and immediately bound to the hydroxyl group of the substrate (Figure 7, 2b followed by 3a). Alternatively, a similar mechanism could allow the formation of a positively charged intermediate, either prior to the generation of the alcohol intermediate (Figures 7, 1 and 2c followed by 3a and 3b) or to form directly the olefinic product (Figures 7, 1 and 2c followed by 3a). The control over the alternative hydroxylation fast step is determined by the orientation of the substrate and the oxidant into the active site. In addition, one of the most important conditions for the desaturation reactions to take place is the presence of an electron transport system, which allows the transfer of an electron from nicotinamide adenine dinucleotide phosphate NAD(P)H cofactor (Figure 8). Particularly, two structurally different, but functionally equivalent, electron donor systems have been identified for FA desaturases.^18^ They have been shown to be specific for the subcellular compartment, where they are localised, and to be a distinctive element between different classes of desaturases: ferredoxin and a cytochrome *b5*‐like unit.^56^ (Figure 8) Ferredoxin, a soluble iron–sulfur protein, is mostly found in plastids of higher plants and in cyanobacteria, and it is used for electron transport by acyl‐ACP and acyl‐lipid desaturases.^57^ (Figure 8a) In plants, this protein has been found in two different isoforms and classified as photosynthetic and heterotrophic according to the tissue specificity and dependence upon light. In the first case, it plays a role of an electron partitioning system in the chloroplasts, by creating a fine balance between photosynthetic processes that involve electron transport and other metabolic reactions such as desaturation reactions.^58^ In fact, extensive characterisation has demonstrated that ferredoxin‐reducing systems are dependent on NADPH/ferredoxin NADP+ reductase (Figure 8a) or on photoreduction of ferredoxin, providing in these two ways electrons for the desaturation reactions. The second isoform has been found to be instead more ubiquitous and independent from light regulation.^59^ It has also been clarified that distinct ferredoxin isoforms are able to influence the production of UFAs, according to their level of activity and the type of desaturase that they interact with.^60^ On the other hand, cytochrome *b*_*5*_, which is a small heme‐binding protein, is usually found in the endoplasmic reticulum of higher plants, animals, yeast and fungi, and it is fundamental for an efficient desaturation of FAs by membrane bounding desaturases, and particularly acyl‐CoA desaturases and some acyl‐lipid desaturases. (Figure 8b)^61^ The electron transfer function is displayed via two possible different mechanisms. One plausible mode of action is related to the desaturation carried out by a desaturases multienzymes complex constituted of desaturases, cytochrome *b*_*5*_ and NADH cytochrome *b*_*5*_ reductase. (Figure 8b) During the process of insertion of the double bond, cytochrome *b*_*5*_ transfers electrons through lateral diffusion in the membrane from NADH cytochrome *b*_*5*_ reductase to the desaturases enzyme.^19^ (Figure 8b) Another possible mechanism accounts for a modular desaturase system made of the desaturase and cytochrome *b*_*5*_ units on the N‐ or C‐ termini of the desaturases.^19^ (Figure 8b) In both cases, the overall process consists of an electron transfer process from NADH cytochrome *b*_*5*_ reductase and FA substrate by cytochrome *b*_*5*_ domain to molecular oxygen, to give an olefinic FA product and two molecules of water, freeing NAD^+^. (Figure 8b)^62^ This type of fusion protein are extremely important for the biosynthesis of PUFAs because they enable the electron transfer to happen faster, without the requirement for an independent cytochrome *b*_*5*_ unit.^63^ It is clear how these two electron transfer systems have a great potential to be genetically engineered in order to structurally reorganise the complex of the electron transport system and desaturase to increase the production of PUFAs in various microorganisms.

## 7 UNUSUAL FATTY ACIDS ARE PRODUCTS OF UNUSUAL DESATURASES

The desaturases that have been described so far are responsible for the biosynthesis of commonly occurring UFAs, which are methylene‐interrupted FAs with double bonds in the *cis* configuration. Together with this group of FAs, more than 1,000 have been found to have unusual chemical structures including non‐methylene‐interrupted PUFAs (NMI‐PUFAs), UFAs with a double bond in a *trans* configuration, or characterised by the presence of functional groups such as hydroxyl, epoxy or cyclopropyl, halogen or acetylenic groups on their acyl chain.^36^ These unusual fatty acids are mostly produced by plants and stored in seed oils, and only very rarely in the membrane glycerophospholipid side chains. Some of them are able to form polymeric structures that compose the external surfaces of plants in many variations.^64^ These findings have therefore identified unusual desaturase enzymes in plants, mostly localised in the endoplasmic reticulum, which is able to produce a very wide variety of FAs with an exceptional structural diversity.^64^ The enzymes able to add unusual functional groups to FAs are identified as a variation of the previously studied desaturases and are classified as bifunctional desaturase/hydroxylase systems.^11^ An example is the castor FA hydroxylase‐12, which transforms PC‐18:1 into PC‐18:1‐OH.^65^ Another example of modified FAs from plants is epoxyoctaenoic FAs produced from PC‐18:1 in *Vernonia galamensis*.^66^ Other divergent FA desaturases have been found to have a conjugase activity, forming trienoic FA alpha‐eleoesterate in *Calendula officinalis*, *Momordica charantia* and *Vernicia fordii*.^67^ The initial hydrogen abstraction in this particular case occurs at C‐11 with the same mechanism described for acyl‐CoA desaturase, but a change in the position of the iron oxidant relative to the substrate has been identified to determine a 1,4‐dehydrogenation‐like reaction.^11^, ^67^ Moreover, a large variety of NMI‐PUFAs, especially the ones containing ∆5‐*cis*‐ethylenic double bond, have been described in plants, animals and primitive low organisms, such as taxoleic acid (∆^5,9^‐18:2), pinolenic acid (∆^5,9,12^‐18:3), sciadonic acid (∆^5,11,14^‐20:3) and juniperonic acid (∆^5,11,14,17^‐20:4).^68^ Recently two desaturases have been identified in *A. leveillei* and defined as Acyl‐CoA‐dependent desaturases class, because of their structural similarity with ∆9‐stearoyl‐CoA‐desaturase.^69^ Exceptionally, a cytochrome *b*_5_ fusion ω3‐desaturase has been discovered in *Chlamydomonas reinhardtii*, which is able to produce C20 NMI‐PUFAs, instead of the more typical methylene interrupted arachidonic acid.^70^ These are only a few examples of various and complex desaturase systems that are able to produce a wider variety of UFAs. These molecules have recently received increased interest from both the pharmaceutical and biofuel industries.

## 8 EXPRESSION AND REGULATION OF DESATURASES GENES

Microorganisms, plants and animals are able to finely regulate the biosynthesis of UFAs in response to chemical and physical changes within the cell and from the external environment.^71^ The control of lipid homeostasis is obtained by the activation of feedback regulation cascades that control the level of expression of genes encoding for FA desaturases by the activation of integral membrane sensors.^71^ Plant desaturases represent one of the most remarkable examples of the regulation of desaturase genes expression, whose level can variate according to changes in phytohormone levels or in exposure to abiotic (temperature, light, osmosis, etc.) and biotic (phytopathogens) factors.^72^ Temperature has been demonstrated to be one of the most effective physical parameters to be investigated: plants showed great ability in regulating the desaturase gene expression according to the variation of temperature in order to maintain a balanced fluidity in the membrane.^73^ In fact, thanks to a wide number of investigations, it was established that the expression of desaturase genes increases in response to exposure to a lower temperature.^72^ An example of this regulation process was found in the study on the desaturases from *Medicago truncatula* genes for FAD3 (fatty acid desaturase) and FAD7 desaturases where an increase in the gene encoding for these desaturases was detected when the plants were grown at a lower temperature.^74^ Variation of the concentration of the salts and the osmotic pressure in the cell environment also generates a response in desaturase expression regulation. Particularly, it has been established that a higher level of salts is able to change the UFA profile in some plants, because of an induced decrease in gene expression. In *Arachis hypogaea*, the exposure to a high concentration of NaCl has resulted in a reduced expression of the genes encoding for ω3‐desaturases, and consequently, a lower level of α‐linolenic acid was also detected.^75^ Temperature variations are also able to change UFA composition of plant membrane lipids via activation of post‐transcriptional and post‐translational modifications.^72^ More specifically, exposure to low temperature increases the mRNA level for desaturases and therefore their expression and level of UFA products.^76^ In a study about FAD3 in *A. thaliana*, post‐translational modifications have also been identified to have an effect on the biosynthesis of UFAs. It has been shown that when either the 5′‐UTR or N‐terminal regions of the desaturase gene sequences were enhanced in the number of copies, the expression of the desaturases would consequently increase.^77^ On the other hand, for FAD8 in *A. thaliana*, the increase of the temperature can activate the opposite mechanism on the C‐terminal of the desaturase gene sequence, with a consequent reduction of production of UFAs.^78^ Experimental data on FAD2 from *A. thaliana* has shown that the gene expression of plants desaturases may also be regulated according to concentration and tissue‐specificity of exposure to chemicals and phytopheromones and consequently suggesting that desaturases are involved in plant signal transduction for important mechanisms of cell adaptation.^79^ Pathogens and injuries are other factors able to trigger the plant cells’ response via increased expression of desaturases, such as the one able to produce α‐linolenic acid, which is the precursor of jasmonic acid, well known to be involved in mechanisms of plant tissues protection.^80^ Plants are the best example of fine regulation of desaturase gene expression, but other mechanisms have been identified also for desaturase pools of various microorganisms and animals. Thus, it has been demonstrated that the addition of FA substrates in the culture media of yeast like *S. cerevisiae* has led to reduced activity of stearoyl‐CoA desaturases and that the same feedback regulation for gene expression has been also found in animals.^81^, ^82^ As well as for plants, also in animals and particularly fishes, the exposure to temperature variation determines the upregulation of acyl‐CoA desaturases. Moreover, this class of desaturases have been found to be finely regulated via post‐translational mechanisms. Particularly, the expression of Δ9 and Δ6‐acyl‐CoA desaturases is controlled by hormones, such as insulin, in animals.^83^ Exceptional mechanisms of transcriptional regulation of the expression of FA desaturases have been studied for ∆5 desaturases in the cyanobacteria *B. subtilis*.^84^ This organism is able to activate a two‐component system made of a histidine kinase and its response regulator for the expression of FA desaturases in the membrane.^84^ The kinase temperature sensor auto‐phosphorylates on the cytoplasmic C‐terminal in response to membrane alteration due to temperature changes.^84^ The phosphate group is then transferred to the desaturase regulator, which interacts with the ∆5 desaturase's promoter increasing the recruitment of RNA polymerases and, consequently, the downstream expression of the desaturase.^84^ Animal cells have been found to account for the regulation system defined sterol regulatory element‐binding pathway (SREBP) which is able to finely control the level of expression of ∆9, ∆5 and ∆6 desaturases.^85^, ^86^ The transcriptional regulation is controlled via a signalling pathway of RNA polymerase I (RIP), where the cleavage of SERBP activates a sterol cleavage activation protein (SCAP) in the endoplasmic reticulum, which then interacts with the sterol‐sensing domain for the regulation of synthesis and uptake of FAs.^85^, ^86^

Through this description, it is clear how biosynthesis of UFAs catalysed by desaturases is a very complex and finely regulated process. This accounts for a coordinated system of feedback regulation of transcription in all organisms, that allows for the maintenance of cell membrane homeostasis and therefore biological functions.^71^

## 9 DESATURASES BIOTECHNOLOGICAL APPLICATION FOR PUFAS PRODUCTION

The broader knowledge around the structure–activity relationship for FA desaturases, the mode of action, the substrate specificity and selectivity and the variability of products constitute a solid base to develop new biotechnological advances in the synthesis of UFAs.^24^ The synthesis of PUFAs has been given particular attention, because of the essential roles that PUFAs play in physiological functions and human health. The interest in fully comprehending the biosynthetic pathway of PUFAs has increased lately in the biotechnological field.^25^ Why is this aspect so important to science? The answer can be found in the high biological value and industrial potential of these biomolecules, and the consequent constantly increasing world demand, which is not correspondently met by PUFAs natural sources, such as higher plants, seed oils and fish. In fact, climate change, agribusiness, overfishing, seawater plastics and heavy metals pollution have damaged and diminished the common sources of EPUFAs.^87^ For all of these reasons, researchers have started to explore alternatives to increase and improve the production of PUFAs at lower costs.^88^ To achieve this goal, it has been fundamental to take advantage of the wider and more detailed knowledge around the key role that FA desaturases play in the synthesis of UFAs.^25^ In particular, microorganisms such as microalgae, fungi, yeast and bacteria have shown to be very promising biological systems to be investigated for the production of PUFAs, exclusively focusing on remarkable FA desaturase machineries.^89^ Chemical biology, genetics and metabolic engineering of desaturase genes have been used as efficient tools to produce PUFAs and their products in high yield by developing a whole new range of sustainable and renewable FA sources in form of cell bio‐factories, with great potential for large scale production.^90^, ^91^ There are various examples of bioengineered microorganisms for the biosynthesis of PUFAs: ω‐3 for the synthesis of arachidonic and eicosapentaenoic acids in *Mortierella alpina*; *S. cerevisiae* and *E. coli* have been used as heterologous systems to express and characterise *S. putrefaciens* desaturases for EPA synthesis,^92^ ∆4 from *I. galbana*,^93^ ∆5 from *T. aureum* and *M. alpina*,^94^ ∆12 and ∆15 from *H. polymorpha*.^95^ Furthermore, transgenic plants have also been developed to enhance the production of PUFAs in seed oils: different desaturases, such as ∆9 desaturases from *I. galbana*, ∆8 from *E. gracilis*, ∆5 desaturases from *M. alpina* and more genes encoding ω‐3 desaturases were cloned into *A. thaliana* to increase the yield of PUFA products.^96^ The same technique has been applied to produce transgenic and PUFAs rich linen seeds and safflower seeds by heterologous overexpression respectively of ∆6 desaturases from *P. vialii* and ∆6 and ∆12 desaturases from *M. alpina*.^97^ Interestingly, transgenic systems expressing FA desaturases, and particularly *C. elegans* ω‐3 desaturases, have been studied and used for clinical treatments of colon and breast cancer, skin damage, reproduction, intestine diseases and neuronal apoptosis.^98^, ^99^, ^100^

## 10 CONCLUSION AND PERSPECTIVE

The biosynthesis of UFAs is an essential pathway from prokaryotes to eukaryotes, from microorganisms to plants and animals: it is the way in which all organisms are able to control the lipid membrane homeostasis and a numerous series of biological cell functions. In order to maintain a balance in the pool of UFAs in the cell, a large variety of desaturases are involved in catalysing the insertion of a certain number of double bonds on a FA molecule in a stereospecific and selective manner. Desaturases have been shown to be a remarkable group of enzymes for their structure–function binomial relationship, to display their catalytic activity to synthesise their products, with chemical structure diversity, from the most common UFAs to unusual FAs. The expression of these enzymes is finely modulated by feedback control of transcriptional regulation of their genes from environmental factors. This structural, mechanistic and biochemical information have been gathered after decades of investigation around FA desaturases, which have led to a broader understanding of the importance and functions of the enzymes and their products. Thus, cloning and identification of a large variety of desaturases in a broad number of different organisms have given insights into the PUFA biosynthetic pathway and their importance in many physiological processes. Therefore, it has opened a modern path for lipidomic research to try and achieve one of the biggest tasks in the field: finding alternative sources of PUFAs to meet the high demand for this essential biomolecule in pharmaceutical, nutraceutical and food industries. The challenge has already started with the use of biotechnological tools to develop promising PUFA cell bio‐factories. The process is still open to a large number of improvements that can be achieved in the near future by always bringing new knowledge in the field around the structures and mechanism of desaturase, and by looking at the development of new, more efficient, easily reproducible, sustainable bioengineering advances.

**Abbreviations**
ACP: acyl carrier protein, 1
CoA: coenzyme A, 1
EPUFA: essential polyunsaturated fatty acid, 3
FAD 3: fatty acid desaturase 3, 21
FAs: Fatty acids, 3
NMI‐PUFAs: non‐methylene‐interrupted PUFAs, 20
PUFAs: polyunsaturated fatty acids, 1
RIP: pathway of RNA polymerase I, 23
SCAP: sterol cleavage activation protein, 23
SREBP: sterol regulatory element‐binding pathway, 23
UFAs: unsaturated fatty acids, 1
VLC‐PUFA: very long‐chain polyunsaturated fatty acids, 3
